# Endovascular Thrombectomy for Acute Stroke with a Large Ischemic Core: A Systematic Review and Meta-Analysis of Randomized Controlled Trials

**DOI:** 10.1007/s00062-023-01306-x

**Published:** 2023-05-26

**Authors:** Mohamed Abuelazm, Unaiza Ahmad, Husam Abu Suilik, Amith Seri, Abdelrahman Mahmoud, Basel Abdelazeem

**Affiliations:** 1 Faculty of Medicine, Tanta University, Tanta, Egypt; 2 Punjab Medical College, Faisalabad, Pakistan; 3 Faculty of Medicine, Hashemite University, Zarqa, Jordan; 4 Department of Internal Medicine, McLaren Health Care, Flint, MI USA; 5 Department of Internal Medicine, Michigan State University, East Lansing, MI USA; 6 Faculty of Medicine, Minia University, Minia, Egypt

**Keywords:** Endovascular thrombectomy, Mechanical thrombectomy, Stroke, Thrombosis, Emergency, Review, Meta-analysis

## Abstract

**Background:**

Endovascular thrombectomy (ET) efficacy and safety in stroke with a large ischemic core is still inconclusive as this population has been underrepresented in ET randomized controlled trials (RCTs).

**Methods:**

We conducted a systematic review and meta-analysis synthesizing RCTs, which were retrieved by systematically searching: PubMed, Web of Science, SCOPUS, and Cochrane through February 18th, 2023. Our primary outcome was neurological disability measured by the modified Rankin scale (mRS). Dichotomous outcomes were pooled using risk ratio (RR) along with confidence interval (CI) using Revman V. 5.4 software.

**Results:**

Three RCTs with a total of 1010 patients were included in our analysis. ET significantly increased the rates of functional independence (mRS ≤ 2) (RR: 2.54 with 95% CI [1.85, 3.48]), independent ambulation (mRS ≤ 3) (RR: 1.78 with 95% CI [1.28, 2.48]), and early neurological improvement (RR: 2.46 with 95% CI [1.60, 3.79]). However, there was no difference between endovascular thrombectomy and medical care in excellent neurological recovery (mRS ≤ 1) (RR: 1.35 with 95% CI [0.88, 2.08]). Also, ET significantly reduced the rate of poor neurological recovery (mRS 4–6) (RR: 0.79 with 95% CI [0.72, 0.86]). However, endovascular thrombectomy was associated with more incidence of any intracranial hemorrhage (RR: 2.40 with 95% CI [1.90, 3.01] [0.72, 0.86]).

**Conclusion:**

ET combined with medical care was associated with better functional outcomes compared with medical care alone. However, ET was associated with a higher rate of intracranial hemorrhage. This can support extending ET indication in the management of stroke with a large ischemic core.

**Supplementary Information:**

The online version of this article (10.1007/s00062-023-01306-x) contains supplementary material, which is available to authorized users.

## Introduction

Endovascular thrombectomy (ET) is shifting paradigms in the therapy regimens for acute ischemic stroke (AIS) caused by large vessel occlusion (LVO). Selected patients with LVO have shown better outcomes with ET as compared to medical therapy alone [1–5]. Current guidelines state that ET is considered when the terminal section of the internal carotid artery or the main stem of the middle cerebral artery is blocked [6–8], with ischemic cores on imaging no greater than 70 ml and Alberta Stroke Program Early Computed Tomographic Score (ASPECTS) greater than six, or when there is a disparity between the volume of perfusion delay area and the ischemic core volume [9, 10]. Current guidelines do not recommend ET for patients with low Alberta Stroke Program Early Computed Tomographic Score (ASPECTS < 6) or large ischemic cores (> 70 ml) on imaging because they have been historically underrepresented in thrombectomy trials; hence, evidence in this regard is still limited [11–14].

However, more recently, several meta-analyses based primarily on observational studies have suggested better neurological outcomes and lower death rates in (ASPECTS 0–5) and infarct-core volumes from ≥ 50 mL or greater on CT perfusion or diffusion-weighted magnetic resonance imaging (MRI) [15–18]. Furthermore, the RESCUE-Japan LIMIT randomized controlled trial (RCT) (Recovery by Endovascular Salvage for Cerebral Ultra-Acute Embolism-Japan Large Ischemic Core Trial) from Japan demonstrated that individuals with an ASPECTS value of 3 to 5 had better functional outcomes with endovascular therapy than with medical care. Still, they also experienced more intracranial hemorrhages [19]. This was furtherly supported by the most recent findings from the Chinese ANGEL-ASPECT RCT [20] and the international SELECT‑2 RCT [21], which showed that patients with acute LVO in the anterior circulation and an ASPECTS score of 3–5 and an infarct-core volume of 70 to 100 ml [20]/≥ 50 mL [21] had better outcomes with endovascular therapy administered within 24 h than with medical management alone.

Therefore, the latest evidence mandates an up-to-date review. Our meta-analysis aims to investigate the safety and efficacy of ET & medical therapy versus medical therapy alone in patients with AIS and ASPECTS (3–5).

## Methodology

### Protocol Registration

Our systematic review and meta-analysis adhered to the guidelines provided by the PRISMA statement [22] and the Cochrane handbook for systematic reviews and meta-analyses [23]. The protocol for this review has been registered and published in PROSPERO with the following ID: CRD42023407277.

### Data Sources and Search Strategy

(B.A. and M.A.) performed a thorough electronic search for relevant literature by utilizing several databases, including PubMed (MEDLINE), Web of Science, SCOPUS, and the Cochrane Central Register of Controlled Trials (CENTRAL) until February 18th, 2023. They did not use any limitations on their search. Further details about the search strategy, including the keywords and search terms, as well as the results of the search, can be found in (Table S1).

### Eligibility Criteria

A PICO criterion was used to include RCTs: population (P): patients with AIS with a large infarct size defined as large vessel occlusion with ASPECTS score 3 to 5; intervention (I): endovascular thrombectomy plus medical therapy; control (C): medical therapy alone; outcome (O): primary outcomes of this review are the efficacy outcomes: early neurological improvement assessed by ≥ 4 points reduction in the National Institutes of Health Stroke Scale (NIHSS), excellent neurological recovery (modified Rankin Scale (mRS) 0–1), functional Independence (mRS 0–2), and independent ambulation (mRS 0–3). The secondary outcomes included safety outcomes: (any-cause mortality at 90 days, poor neurological recovery (mRS 4–6), any intracerebral hemorrhage, symptomatic intracerebral hemorrhage, and decompressive craniectomy).

We did not consider a range of research designs in our analysis. Specifically, we excluded non-human studies, preliminary reports, various forms of observational studies, single-arm clinical trials, in vitro experiments conducted on tissues and cultures, book chapters, editorial, and press articles, publications that only contain abstracts or posters, unpublished study protocols, and studies that were conducted in languages other than English.

### Study Selection

The review process was carried out using the Covidence online tool. (H.A.S. and A.S.) reviewed the retrieved records independently after eliminating any duplicated records. The full-texts of the records that met the initial eligibility criteria were examined through full-text screening. Any discrepancy was resolved by consensual discussion and agreement.

### Data Extraction

Two reviewers (H.A.S. and A.S.) independently extracted all data using a standardized electronic spreadsheet: study characteristics (country, study design, total participants, main inclusion criteria, intervention, and comparison methods, ASPECTS, timing after symptoms onset (time window), and baseline imaging); baseline characteristics (age, sex, number of patients in each group, ASPECTS score, NIHSS, infarct core volume, and occlusion location); efficacy outcomes data (early neurological improvement, excellent neurological recovery (mRS 0–1), functional Independence (mRS 0–2), and independent ambulation (mRS 0–3)), and safety outcomes (any-cause mortality at 90 days, poor neurological recovery (mRS 4–6), any intracerebral hemorrhage, symptomatic intracerebral hemorrhage, and decompressive craniectomy). Any discrepancy was resolved by consensual discussion and agreement.

### Risk of Bias and Quality Assessment

Two reviewers (H.A.S. and A.S.) assessed the quality of the studies included in the research independently using the Cochrane RoB2 tool [24]. The domains that were evaluated included the risk of bias resulting from the randomization process, the risk of bias due to deviation from the intended intervention, the risk of bias due to missing outcome data, the risk of bias in measuring the outcome, and the risk of bias in selecting the reported results. In the event of any disagreements, the reviewers discussed and resolved them through consensus.

To appraise the quality of evidence, two reviewers (M.A. and B.A.) utilized the Grading of Recommendations Assessment, Development, and Evaluation (GRADE) guidelines [25, 26]. The evaluation was carried out for each outcome, and the decisions were justified and documented. Any discrepancy was resolved by consensual discussion and agreement.

### Statistical Analysis

The RevMan v5.3 software [27] was used for statistical analysis. To combine the outcomes for dichotomous outcomes, the risk ratio was used, while the mean difference (MD) was used for continuous outcomes. Both were calculated with a 95% confidence interval (CI) using the fixed-effects model. However, the random-effects model was used in case of significant heterogeneity. The presence and extent of heterogeneity were evaluated using the Chi-square and I‑square tests, respectively. Following the Cochrane Handbook (chapter nine) [28], heterogeneity was considered significant if the alpha level for the Chi-square test was below 0.1, while the I‑square test results were interpreted as follows: not significant for 0–40%, moderate heterogeneity for 30–60%, and substantial heterogeneity for 50–90%. On significant heterogeneity, a leave-one-out sensitivity analysis was conducted.

## Results

### Search Results and Study Selection

**Fig. 1:**
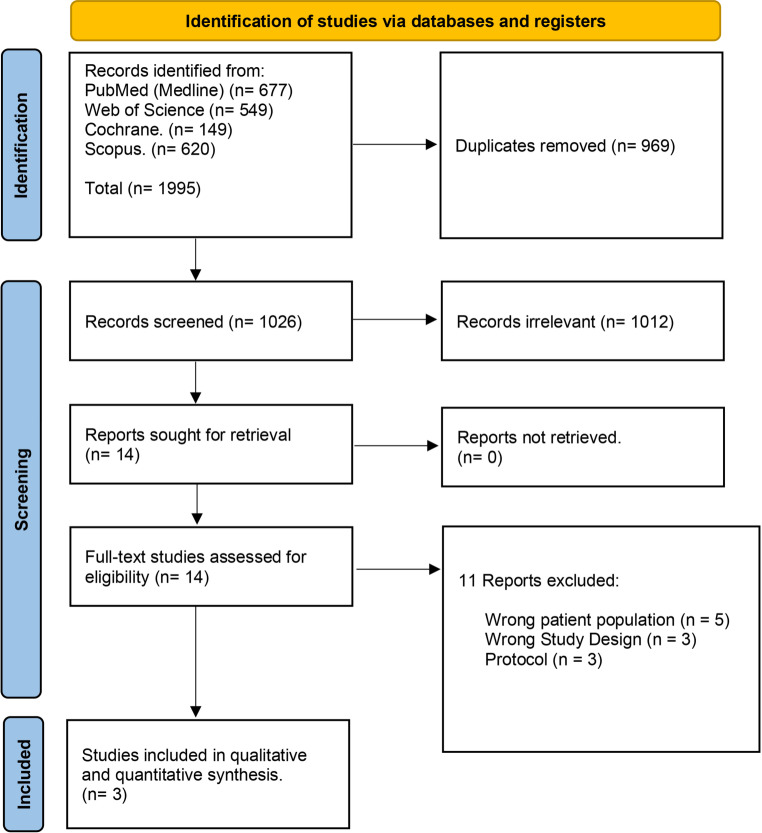
PRISMA flow chart of the screening process

A total of 1995 studies were identified and evaluated for screening based on their titles and abstracts. After removing 969 duplicates and 1012 studies that did not match the inclusion criteria, fourteen full-text articles were assessed. Out of these, eleven were found to be irrelevant and excluded, leaving three RCTs to be included in the qualitative and quantitative analysis (Fig. 1).

### Characteristics of Included Studies

We included a total of three RCTs [19–21]. Detailed summary characteristics of the included studies are outlined in (Table 1). A total of 1010 patients were included, of which 509 were allocated to the ET group and 501 patients to the medical management group. Most patients were men, including 297 (58.3%) in the ET group and 302 (60.3%) in the medical management group. Further baseline characteristics are highlighted in (Table 2).

**Table 1:** Summary characteristics of the included RCTs

Study ID	Study Design	Country	Total Participants	Interventions		Main Inclusion Criteria	Baseline Imaging
				ET Method	Medical Care		
Huo et al. (2023) (ANGEL-ASPECT) [20]	Multicenter open-label RCT	China	455	Stent retriever or contact aspiration	Alteplase (0.9 mg/kg body wgt. Or Urokinase (1 to 1.5 million IU))	Age 18 to 80 years, AIS within previous 24 hrs with NIHSS 6 to 30, large vessel occlusion, ASPECTS 3–5, and LIC 70–100 ml	CTA, MRA
Sarraj et al. (2023) (SELECT2) [21]	Multicenter open-label RCT	USA, Canada, Europe, Australia, and New Zealand	352	Stent retriever, aspiration devices or both	Tenecteplase or Alteplase	Age 18 to 85, AIS within previous 24 hrs due to occlusion of ICA or M1 MCA, ASPECTS 3–5, and LIC ≥ 50 ml	MRI, non-contrast CT
Yoshimura et al. (2022) (RESCUE-Japan LIMIT) [19]	Multicenter open-label RCT	Japan	203	Stent retriever, aspiration catheter, balloon angioplasty, intracranial stent, or carotid-artery stent	Alteplase (0.6 mg/kg)	> 18 years, AIS within previous 24 hrs due to occlusion of the ICA or M1 MCA, ≥ 6 on NIHSS, mRS 0 or 1 before AIS, and ASPECTS 3–5	MRI, non-contrast CT

*RCT* randomized controlled trial, *ET* endovascular thrombectomy, *AIS* acute ischemic stroke, *hrs* hours, *NIHSS* National Institutes of Health Stroke Scale, *mRS* modified Rankin Scale, *ASPECTS* Alberta Stroke Program Early Computed Tomographic Score, *LIC* large infarct core

**Table 2:** Baseline characteristics of the participants

Study ID	Number of Patients		Age (Years) Mean (SD)		Gender (Male) N. (%)		ASPECTS, median (IQR)		NIHSS, median (IQR)		Infarct Core Volume, median (IQR)		Occlusion Location N. (%)					
	ET	MC	ET	MC	ET	MC	ET	MC	ET	MC	ET	MC	ICA		M1 Segment		M2 Segment	
													ET	MC	ET	MC	ET	MC
Huo et al. (2023) (ANGEL-ASPECT) [20]	230	225	67.3 (8.9)	66.3 (10.4)	135 (58.7)	144 (64)	3 (3–4)	3 (3–4)	16 (13–20)	15 (12–19)	60.5 (29–86)	63 (31–86)	83 (36.1)	81 (36)	145 (63)	142 (63.1)	2 (0.9)	2 (0.9)
Sarraj et al. (2023) (SELECT2) [21]	178	174	66.3 (12.7)	66.6 (12.7)	107 (60.1)	100 (57.5)	4 (4–5)	4 (4–5)	19 (15–23)	19 (15–22)	81.5 (57–118)	79 (62–111)	80 (44.9)	66 (37.9)	91 (51.1)	100 (57.5)	7 (3.9)	8 (4.6)
Yoshimura et al. (2022) (RESCUE-Japan LIMIT) [19]	101	102	76.6 (10)	75.7 (10.2)	55 (54.5)	58 (56.9)	3 (3–4)	4 (3–4)	22 (18–26)	22 (17–26)	94 (66–152)	110 (74–140)	47 (46.5)	49 (48)	74 (73.3)	70 (68.6)	0	3 (2.9)

*SD* standard deviation, *N.* number, *IQR* interquartile range, *ET* endovascular thrombectomy, *NIHSS* National Institutes of Health Stroke Scale, *ASPECTS* Alberta Stroke Program Early Computed Tomographic Score

### Risk of Bias and Quality of Evidence

**Fig. 2:**
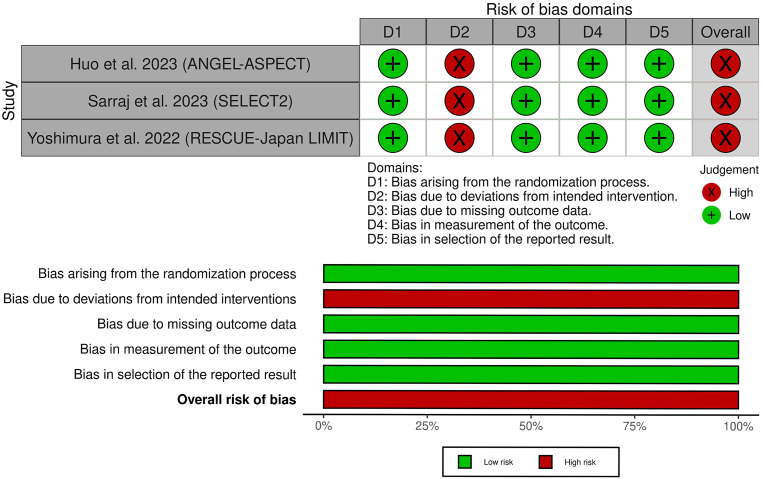
Quality assessment of the risk of bias in the included trials. The upper panel presents a schematic representation of risks (low = *red*, unclear = *yellow*, and high = *red*) for specific types of biases of each of the studies in the review. The lower panel presents risks (low = *red*, unclear = *yellow*, and high = *red*) for the subtypes of biases of the combination of studies included in this review

Using the Cochrane RoB2 tool’s five domains, we evaluated each outcome included in the quantitative synthesis’s risk of bias (Fig. 2). All of the included RCTs showed an overall high risk of bias, mainly attributed to the performance bias due to the lack of blinding.

### Efficacy Outcomes

**Fig. 3:**
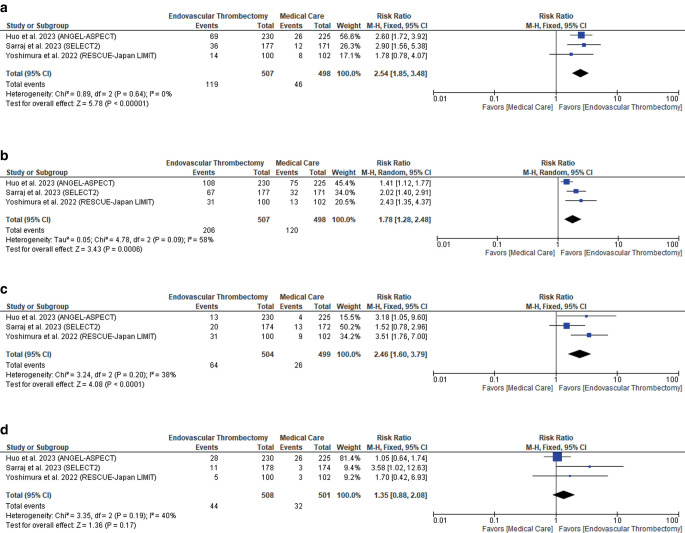
Forest plot of the efficacy outcomes. **a** Functional independence (mRS ≤ 2), **b** independent ambulation (mRS ≤ 3), **c** early neurological improvement, **d** excellent neurological recovery (mRS ≤ 1). (*RR* risk ratio, *CI* confidence interval)

Endovascular thrombectomy significantly increased the rates of functional independence (mRS ≤ 2) (RR: 2.54 with 95% CI [1.85, 3.48], *P* = 0.00001) (low-quality evidence) (Fig. 3a; Table 3), independent ambulation (mRS ≤ 3) (low-quality evidence) (RR: 1.78 with 95% CI [1.28, 2.48], *P* = 0.0006) (low-quality evidence) (Fig. 3b; Table 3), and early neurological improvement (RR: 2.46 with 95% CI [1.60, 3.79], *P* = 0.0001) (low-quality evidence) (Fig. 3c; Table 3). However, there was no difference between endovascular thrombectomy and medical care in excellent neurological recovery (mRS ≤ 1) (RR: 1.35 with 95% CI [0.88, 2.08], *P* = 0.17) (low-quality evidence) (Fig. 3d; Table 3).

**Table 3:** GRADE evidence profile

Certainty assessment							Summary of findings				
Participants (studies) Follow-up	Risk of bias	Inconsistency	Indirectness	Imprecision	Publication bias	Overall certainty of evidence	Study event rates (%)		Relative effect (95% CI)	Anticipated absolute effects	
							With [comparison]	With [intervention]		Risk with [comparison]	Risk difference with [intervention]
*Functional Independence (mRS 0–2)*											
1005 (3 RCTs)	Serious^a^	Not serious	Not serious	Serious^b^	None	⨁⨁◯◯ Low	46/498 (9.2%)	119/507 (23.5%)	**RR 2.54** (1.85 to 3.48)	92 per 1000	**142 more per 1000** (from 79 more to 229 more)
*Independent Ambulation (mRS 0–3)*											
1005 (3 RCTs)	Serious^a^	Serious^c^	Not serious	Not serious	None	⨁⨁◯◯ Low	120/498 (24.1%)	206/507 (40.6%)	**RR 1.78** (1.28 to 2.48)	241 per 1000	**188 more per 1000** (from 67 more to 357 more)
*Early Neurological Improvement*											
1003 (3 RCTs)	Serious^a^	Not serious	Not serious	Serious^b^	None	⨁⨁◯◯ Low	26/499 (5.2%)	64/504 (12.7%)	**RR 2.46** (1.60 to 3.79)	52 per 1000	**76 more per 1000** (from 31 more to 145 more)
*Excellent Neurological Recovery (mRS 0–1)*											
1009 (3 RCTs)	Serious^a^	Not serious	Not serious	Serious^b^	None	⨁⨁◯◯ Low	32/501 (6.4%)	44/508 (8.7%)	**RR 1.35** (0.88 to 2.08)	64 per 1000	**22 more per 1000** (from 8 fewer to 69 more)
*Poor Neurological Recovery (mRS 4–6)*											
1009 (3 RCTs)	Serious^a^	Not serious	Not serious	Serious^d^	None	⨁⨁◯◯ Low	378/501 (75.4%)	301/508 (59.3%)	**RR 0.79** (0.72 to 0.86)	754 per 1000	**158 fewer per 1000** (from 211 fewer to 106 fewer)
*ALL-Cause Mortality at 90 days*											
1005 (3 RCTs)	Serious^a^	Not serious	Not serious	Serious^b^	None	⨁⨁◯◯ Low	140/498 (28.1%)	136/507 (26.8%)	**RR 0.95** (0.78 to 1.16)	281 per 1000	**14 fewer per 1000** (from 62 fewer to 45 more)
*Any Intracranial Haemorrhage*											
657 (2 RCTs)	Serious^a^	Serious^c^	Not serious	Serious^b^	None	⨁◯◯◯ Very low	71/327 (21.7%)	171/330 (51.8%)	**RR 2.30** (1.50 to 3.51)	217 per 1000	**282 more per 1000** (from 109 more to 545 more)
*Symptomatic Intracranial Haemorrhage*											
1009 (3 RCTs)	Serious^a^	Not serious	Not serious	Serious^b^	None	⨁⨁◯◯ Low	13/501 (2.6%)	24/508 (4.7%)	**RR 1.83** (0.95 to 3.55)	26 per 1000	**22 more per 1000** (from 1 fewer to 66 more)
*Decompressive Craniectomy*											
657 (2 RCTs)	Serious^a^	Serious^c^	Not serious	Serious^b^	None	⨁◯◯◯ Very low	22/327 (6.7%)	27/330 (8.2%)	**RR 1.22** (0.43 to 3.41)	67 per 1000	**15 more per 1000** (from 38 fewer to 162 more)

*CI* confidence interval, *RR* risk ratio

^a^All of the included RCTs showed an overall high risk of bias mainly attributed to the performance bias due to the lack of blinding

^b^Number of events is less than 300 event

^c^I^2^ > 50%

^d^The confidence interval does not exclude the risk of appreciable benefit/harm

Pooled studies were homogenous in functional independence (mRS ≤ 2) (*P* = 0.64, I^2^ = 0%), early neurological improvement (*P* = 0.20, I^2^ = 38%), and excellent neurological recovery (mRS ≤ 1) (*P* = 0.19, I^2^ = 40%). However, studies were heterogenous in independent ambulation (mRS ≤ 3) (*P* = 0.09, I^2^ = 58%). Therefore, we performed a sensitivity analysis, and heterogeneity was best resolved by excluding ANGEL-ASPECT RCT (P = 0.60, I^2^ = 0%) (Table S2).

### Safety Outcomes

**Fig. 4:**
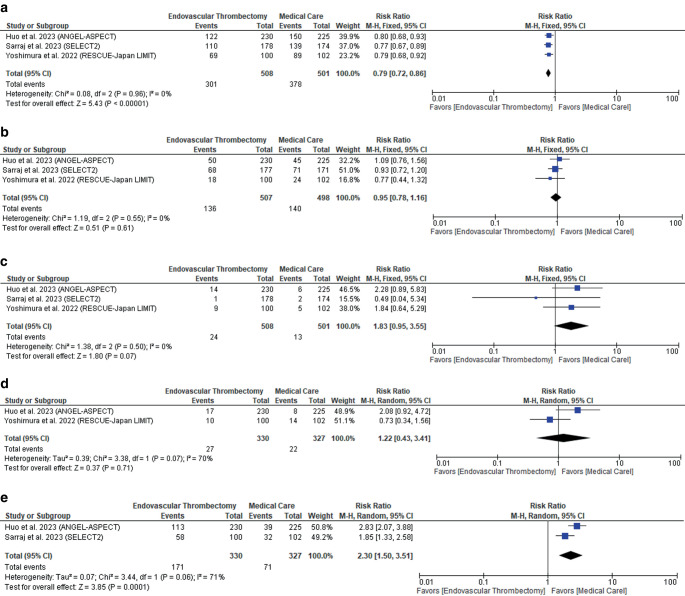
Forest plot of the safety outcomes. **a** Poor neurological recovery (mRS 4–6), **b** all-cause mortality, **c** symptomatic intracranial hemorrhage, **d** decompressive craniectomy, **e** any intracranial hemorrhage. (*RR* risk ratio, *CI* confidence interval)

Endovascular thrombectomy significantly reduced the rate of poor neurological recovery (mRS 4–6) (RR: 0.79 with 95% CI [0.72, 0.86], *P* = 0.00001) (low-quality evidence) (Fig. 4a; Table 3), with no difference regarding all-cause mortality (RR: 0.95 with 95% CI [0.78, 1.16], *P* = 0.61) (low-quality evidence) (Fig. 4b; Table 3), symptomatic intracranial hemorrhage (RR: 1.83 with 95% CI [0.95, 3.55], *P* = 0.07) (low-quality evidence) (Fig. 4c; Table 3), and decompressive craniectomy (RR: 1.22 with 95% CI [0.43, 3.41], *P* = 0.71) (very low-quality evidence) (Fig. 4d; Table 3). However, endovascular thrombectomy was associated with more incidence of any intracranial hemorrhage (RR: 2.30 with 95% CI [1.50, 3.51], *P* = 0.0001) (very low-quality evidence) (Fig. 4e; Table 3).

Pooled studies were homogenous in poor neurological recovery (mRS 4–6) (*P* = 0.96, I^2^ = 0%), all-cause mortality (*P* = 0.55, I^2^ = 0%), and symptomatic intracranial hemorrhage (*P* = 0.50, I^2^ = 0%). However, studies were heterogenous in decompressive craniectomy (*P* = 0.07, I^2^ = 70%) and any intracranial hemorrhage (*P* = 0.06, I^2^ = 71%), and sensitivity analysis was not applicable.

## Discussion

Our meta-analysis showed that ET increased rates of functional independence (mRS ≤ 2), independent ambulation (mRS ≤ 3), and early neurological improvement. However, excellent neurological recovery (mRS ≤ 1) did not differ significantly between the two groups. Similarly, among the safety outcomes ET significantly reduced the rate of poor neurological recovery (mRS 4–6). Other safety outcomes, such as all-cause mortality, symptomatic intracranial hemorrhage, and decompressive craniectomy, were similar between the two groups. Despite the encouraging results, ET significantly increased the risk of any intracranial hemorrhage incidence.

Our results are consistent with the previous evidence, reporting a decreased incidence of unfavorable outcomes in patients treated with ET. In an individual data pooled meta-analysis, Román et al. showed a comparable rise in functional independence rates (mRS-scores 0–2) with ET in patients with ASPECTS less than six or ischemic core volume greater than or equal to 50 ccs or both [17]. Kerleroux et al. performed a meta-analysis on patients who had a substantial ischemic volume at admission and were undergoing ET; the results showed a significant drop in mRS 3–6 [15]. Based on the culminating evidence, it is safe to suggest that endovascular thrombectomy is emerging as an effective intervention as opposed to medical therapy only, even in subjects having large ischemic core volumes.

Without clear guidelines, this opens new hopes for treatment in patients, especially with low ASPECT scores (< 5) and large ischemic cores that have been traditionally factored out from ET trials. Worth noting, previous RCTs intended to demonstrate large treatment effect size and therefore only enlisted patients with small infarct size or ASPECTS 6–10 rigidly defined by imaging techniques [12] and leaving a considerable population of patients who could have profited from the treatment but could not qualify for the imaging inclusion criteria.

Similarly, a higher incidence of mortality or symptomatic intracranial hemorrhage was not reported. Nonetheless, in patients receiving ET, the risk of any intracranial hemorrhage continues to be considerable. However, this outcome did not make ET inferior as a treatment option since symptomatic intracranial hemorrhage risk was not elevated, and it remained to be assessed whether it was the result of a procedural complication or the intervention (ET) itself.

Individual risk for cerebral hemorrhage is influenced by several variables, including age, race, ethnicity, blood pressure control during ET, and the existence of concomitant conditions. Also, it depends on the technique used to retrieve the stent, how long the surgery takes, and how many times it is attempted before successful recanalization. Hence, the safety profile of ET can be further enhanced in the future by using modern imaging techniques to forecast the risk for symptomatic intracranial hemorrhage, such as DW MRI, perfusion CT, and digital subtracted angiography (DSA) for each individual [29].

### Strengths and Limitations

Our meta-analysis is based on data from the three most recent RCTs, with minimal statistical heterogeneity (any heterogeneity encountered was resolved by sensitivity analysis) among outcomes guaranteeing the relevance and reliability of our conclusions to be the gold standard evidence regarding this matter. Nevertheless, there are however certain limitations due to the inherent characteristics of the included studies: first, our results might not be generalizable to all patient populations due to two of the included studies being based in Asian geography (Japan & China) [19, 20]. Second, there is also variability in the time window the patients were enrolled, which can influence patient outcomes. As in ANGEL-ASPECT [20], 63.3% of the patients were enrolled in the 6‑to-24-hour time window, whereas in RESCUE-Japan LIMIT [19], 28.6% of the patients were enrolled in this late window. Third, due to the lack of agreement on the management between different centers, several confounding variables may have gone unreported & therefore impacted our results. Similarly, there may be institutional variability in assessing AIS depending on whether CT or diffusion-weighted MRI was used to calculate ASPECTS values. Fourth, another factor that can introduce bias and hence influence the validity is the difference in group sizes notably, the number of patients receiving IV thrombolysis remained smaller than ET, possibly due to eligibility limitations. Finally, the GRADE assessment yielded low to very low-quality evidence, limiting the clinical endorsement of our findings.

### Implications for Future Research

To address these limitations, First, large-scale RCTs should be conducted, including patients with diverse characteristics: demographic, comorbidities, and stroke risk factors. Studies should be designed to explore patient populations with low ASPECT scores < 5, baseline NIHSS score, large ischemic core, and optimal time window for ET. Further imaging protocols should be standardized by using a specific imaging modality, such as CT or DW-MRI, for stroke assessment. In this regard, results from ongoing RCTs are anticipated to provide promising results. In this regard, ongoing RCTs are committed to exploring the horizons of ET based on varying imaging modalities and inclusion criteria. Results from the European (TENSION, NCT03094715) trial investigating ASPECTS 3–5 at baseline in the extended time window of up to 12 h, the French (IN-EXTREMIS-LASTE, NCT03811769) trial exploring ET in a seven-hour time window, ASPECTS 0–5 on DWI-MRI or non-contrast CT, and the North American (TESLA, NCT03805308) assessing moderately large infarct volume NCCT ASPECTS 2–5 are likely to provide conclusive evidence.

Second, a patient-level & subgroup analysis is warranted for an in-depth exploration of patient factors to determine if patients with certain characteristics are more likely to benefit from ET. Additionally, a long-term follow-up duration, i.e., 12 months in contrast to the typical three months, should be conducted to better assess the impact of ET on patients’ quality of life vs. medical therapy alone. Finally, for ET to be adopted in widespread clinical practice, a cost-effectiveness analysis should be conducted between the two interventions. This is especially important in low and middle-income countries (LMIC) settings where the best evidence is required to convince policymakers and stakeholders to sponsor treatments.

## Conclusion

Overall, ET with routine medical care in patients with AIS with a large ischemic core, defined as ASPECTS 3–5, was associated with better functional outcomes compared with medical care alone. Nonetheless, the rate of intracranial hemorrhage was significantly higher in patients undergoing ET; however, not symptomatic. This can support extending ET indication in the management of AIS with a large ischemic core.

## Supplementary Information

The supplementary material includes: table S1: Search strategy, and table S2: Sensitivity analysis
