# Supplementary material for: Endovascular Thrombectomy for Acute Stroke with a Large Ischemic Core: A Systematic Review and Meta-Analysis of Randomized Controlled Trials
Source: Clin Neuroradiol. 2023 May 26;33(3):625–34. doi: 10.1007/s00062-023-01306-x (PMC10450014; doi:10.1007/s00062-023-01306-x)
Supplement: Supplementary file 1 — The supplementary material includes: table S1: Search strategy, and table S2: Sensitivity analysis [file 62_2023_1306_MOESM1_ESM.docx]

**Supplementary material:**

**Title.**

**Endovascular Thrombectomy for Acute Stroke with a Large Ischemic Core: A Systematic Review and Meta-Analysis of Randomized Controlled Trials.**

**Running Title.**

Endovascular Thrombectomy for Large Stroke.

**Keywords.**

Endovascular thrombectomy, mechanical thrombectomy; stroke; thrombosis; emergency; review; meta-analysis.

**Contents:**

**Tables.**Table S1: Search strategy.

Table S2: Sensitivity analysis.

| Database | Search Terms | Search Field | Search Results |
| --- | --- | --- | --- |
| Pubmed | ("mechanical thrombectomy" OR "endovascular*") AND ("ischemic core" OR "large infarct*" OR "low ASPECTS" OR "large baseline infarct*" OR "large core*" OR "core volume" OR "Diffusion-Weighted Imaging Stroke Lesions" OR "Imaging Lesions" OR "Alberta Stroke Program Early Computed Tomography Score" OR "massive cerebral infarction" OR "large hemispheric infarction" OR "malignant middle cerebral artery infarction") | All Field | 677 |
| Cochrane | ("mechanical thrombectomy" OR "endovascular*") AND ("ischemic core" OR "large infarct*" OR "low ASPECTS" OR "large baseline infarct*" OR "large core*" OR "core volume" OR "Diffusion-Weighted Imaging Stroke Lesions" OR "Imaging Lesions" OR "Alberta Stroke Program Early Computed Tomography Score" OR "massive cerebral infarction" OR "large hemispheric infarction" OR "malignant middle cerebral artery infarction") | All Field | 149 |
| WOS | ("mechanical thrombectomy" OR "endovascular*") AND ("ischemic core" OR "large infarct*" OR "low ASPECTS" OR "large baseline infarct*" OR "large core*" OR "core volume" OR "Diffusion-Weighted Imaging Stroke Lesions" OR "Imaging Lesions" OR "Alberta Stroke Program Early Computed Tomography Score" OR "massive cerebral infarction" OR "large hemispheric infarction" OR "malignant middle cerebral artery infarction") | All Fields | 549 |
| Scopus | ("mechanical thrombectomy" OR "endovascular*") AND ("ischemic core" OR "large infarct*" OR "low ASPECTS" OR "large baseline infarct*" OR "large core*" OR "core volume" OR "Diffusion-Weighted Imaging Stroke Lesions" OR "Imaging Lesions" OR "Alberta Stroke Program Early Computed Tomography Score" OR "massive cerebral infarction" OR "large hemispheric infarction" OR "malignant middle cerebral artery infarction") | TITLE-ABS-KEY | 620 |

*Table S1: Search Strategy.*

| Outcome | Number of  participants (ET/Medical therapy) | No. of  trials | Quantitative data synthesis | | | | Heterogeneity analysis | | |
| --- | --- | --- | --- | --- | --- | --- | --- | --- | --- |
|  |  |  | RR | 95% CI | Z value | p-value | df | p-value | I2 (%) |
| \| **Independent Ambulation (mRS 0–3)** \| \| --- \| | | | | | | | | | |
| All studies | 507/498 | 3 | 1.68 | [1.40, 2.03] | 5.49 | 0.00001 | 2 | 0.09 | 58 |
| Omitting  Huo et al. 2023 (ANGEL-ASPECT) | 277/273 | 2 | 2.14 | [1.57, 2.92] | 4.81 | 0.0001 | 1 | 0.6 | 0 |
| Omitting  Sarraj et al. 2023 (SELECT2) | 330/327 | 2 | 1.56 | [1.26, 1.93] | 4.03 | 0.0001 | 1 | 0.09 | 66 |
| Omitting  Yoshimura et al. 2022 (RESCUE-Japan LIMIT) | 407/396 | 2 | 1.59 | [1.31, 1.94] | 4.67 | 0.00001 | 1 | 0.1 | 64 |

*Table S2: Sensitivity analysis.*
